# Functional Expression of Multidrug Resistance Protein 4 MRP4/ABCC4

**DOI:** 10.1177/2472555219867070

**Published:** 2019-08-05

**Authors:** David Hardy, Roslyn M. Bill, Anass Jawhari, Alice J. Rothnie

**Affiliations:** 1 Life & Health Sciences, Aston University, Birmingham, UK; 2 CALIXAR, Lyon, France

**Keywords:** ABC transporter, membrane protein expression, fluorescence, vesicular transport assay

## Abstract

To study the function and structure of membrane proteins, high quantities of pure and stable protein are needed. One of the first hurdles in accomplishing this is expression of the membrane protein at high levels and in a functional state. Membrane proteins are naturally expressed at low levels, so finding a suitable host for overexpression is imperative. Multidrug resistance protein 4 (MRP4) or ATP-binding cassette subfamily C member 4 (ABCC4) is a multi-transmembrane protein that is able to transport a range of organic anionic compounds (both endogenous and xenobiotic) out of the cell. This versatile transporter has been linked with extracellular signaling pathways and cellular protection, along with conferring drug resistance in cancers. Here we report the use of MRP4 as a case study to be expressed in three different expression systems: mammalian, insect, and yeast cells, to gain the highest yield possible. Interestingly, using the baculovirus expression system with *Sf*9 insect cells produced the highest protein yields. Vesicular transport assays were used to confirm that MRP4 expressed in *Sf*9 was functional using a fluorescent cAMP analogue (fluo-cAMP) instead of the traditional radiolabeled substrates. MRP4 transported fluo-cAMP in an ATP-dependent manner. The specificity of functional expression of MRP4 was validated by the use of nonhydrolyzable ATP analogues and MRP4 inhibitor MK571. Functionally expressed MRP4 in *Sf*9 cells can now be used in downstream processes such as solubilization and purification in order to better understand its function and structure.

## Introduction

One of the limitations of membrane protein structural biology is expressing the membrane protein of interest. The challenge lies in not only expressing the protein of interest but also expressing it to a high level in its native conformation(s). Most membrane proteins are naturally expressed in low levels, and so obtaining sufficient amounts of the native membrane proteins to conduct functional and structural studies requires large amounts of resources and is really only realistic for proteins that are naturally abundant in certain cell types, such as rhodopsin in the retina.^1^

To overcome the problem of low natural expression, recombinant overexpression can be performed, increasing the yield per cell.^2^ Another advantage of recombinant expression is the ability to easily add tags to enable efficient separation of the target protein from the other membrane proteins. Common purification tags include histidine, strep, and flag tags, which can increase the purity and yield though affinity purification.^3^ However, it is important that these tags do not interfere with the function of the protein. Recombinant membrane protein expression is also a means of producing more stable membrane proteins through the use of mutagenesis and protein engineering, but the native conformation will be altered and therefore the correct function and structure will not be discovered.^4^

Effective recombinant membrane protein expression requires finding a suitable host. If the membrane protein is a prokaryotic protein, then *Escherichia coli* could potentially be used. The advantages of using *E. coli* for recombinant overexpression of membrane proteins is that it can be carried out quickly, as *E. coli* have a high growth rate, high quantities of cells are easily achieved, and it is cost-effective.^5^ If the target protein is eukaryotic, such as human membrane proteins, a eukaryotic host such as yeast, insect, or mammalian cells can be used.

Insect cell expression is a commonly used expression system for recombinant mammalian membrane proteins. It requires the production of a recombinant baculovirus carrying the gene of interest, and infection of insect cells, such as *Spodoptera frugiperda* (*Sf*9), with this virus leads to protein expression.^6^ Inclusion bodies are rarely formed with the baculovirus expression system in insect cells, unlike in *E. coli*.^7^ This system has also been beneficial in the production of multiprotein subunit complexes.^8–10^

Two main strains of yeast have been used for membrane protein expression, *Pichia pastoris* and *Saccharomyces cerevisiae. P. pastoris* requires the integration of the recombinant gene of interest into the yeast genome, allowing a stable strain to be produced, but it is not possible to control the number of copies or location of the recombinant gene. On the other hand, *S. cerevisiae* expression tends to use plasmids containing the gene of interest, similarly to *E. coli*. However, the advantages of using *P. pastoris* are the high cells densities it can grow to, with exceptionally high yields of correctly folded protein, meaning a large amount of recombinant protein can be produced,^11^ which is why *P. pastoris* was chosen for this study.

Mammalian cell expression offers potentially the most relevant cellular environment for human membrane proteins. Two of the most common mammalian cell lines used are human embryonic kidney (HEK) and Chinese hamster ovary (CHO) cells.^6,12^ The HEK cell line was chosen for this study as it has increasingly been used for membrane protein expression.^13^ Proteins expressed in HEK cells are usually fully glycosylated compared with *Sf*9 cells.^8^ HEK cells can be made to overexpress recombinant membrane proteins by producing either transient or stable cell lines.^14^ While transient expression can give considerable batch-to-batch variability, creating stable cells often reduces the expression yield. Thus, transient transfections were utilized in this study.

ATP-binding cassette (ABC) transporters are integral membrane proteins that are found in all types of organisms, from prokaryotes to humans. They utilize energy from ATP binding and hydrolysis to transport a variety of substrates across the biological lipid bilayer.^15^ In humans, the 48 different ABC transporters can be separated into 7 different subfamilies, ABCA–ABCG, of which multidrug resistance protein 4 (MRP4/ABCC4) is part of the C subfamily.^16^

MRP4 can be found in a wide range of cells all over the human body, including blood cells, neurons, testis, ovaries, adrenal glands, prostate tubuloacinar cells, and renal proximal tubule cells.^17^ Endogenously, MRP4 is able to transport substrates that are involved in inflammation, such as prostaglandins and leukotrienes^18^ and cell signaling, including cyclic nucleotides such as cyclic AMP (cAMP) and cyclic GMP (cGMP).^19^ It has also been shown to transport a wide range of drugs and their metabolites, including anticancer, antiviral, and antibiotic molecules.^20^

How MRP4 is able to transport such a wide variety of substrates is not well known. In particular, how it can recognize, bind, and transport both relatively hydrophilic molecules like cAMP and hydrophobic molecules such as bile salts or drugs like methotrexate is unclear. This could be due to the lack of structural knowledge about the transmembrane domains (TMDs) of MRP4, which are responsible for transporting substrates. Therefore, functional and structural studies will help reveal the intricacies of this membrane protein.

In this study, we investigated the functional overexpression of MRP4 by examining which approach gave the best expression yield and then characterized the function with a fluorescent vesicular transport assay (VTA).

## Materials and Methods

### S*f*9 Expression

Expression of the recombinant human MRP4-his_6_ within *Sf*9 cells was conducted using a baculovirus encoding for recombinant MRP4 generated from a pFastBac-MRP4-his_6_ construct as described previously.^21^ Cells were grown in shaker cultures using Insect Xpress media (Lonza, Basel, Switzerland). To find the optimal expression conditions, cells at a density of either 1 or 2 million per milliliter were infected with baculovirus using a multiplicity of infection (MOI) of either 2 or 4, and cells harvested after 24, 48, or 72 h.

### *P. pastoris* Expression

Growth media BMGY (buffered glycerol complex medium) and BMMY (buffered methanol complex medium) were made using 10 g of yeast extract, and 20 g of peptone was dissolved in 700 mL of water and autoclaved. After filter sterilization, 100 mL of 1 M potassium phosphate buffer, pH 6.0 (13.2 mL of 1 M K_2_HPO_4_ and 86.8 mL of 1M K_2_HPO_4_), 100 mL of 10× YNB (13.4% yeast nitrogen base with ammonium sulfate without amino acids), 2 mL of 0.02% biotin, and 100 mL of 10% glycerol for BMGY or 100 mL of 5% methanol for BMMY were added.

The recombinant pPICZαC-MRP4-his_6_ construct was created using a double digest of the pFastBac MRP4-his_6_ plasmid and pPICZαC with EcoRI, followed by ligation of MRP4-his_6_ into the pPICZαC plasmid, at a plasmid-to-insert molar ratio of 1:3, overnight at 16 °C. pPICZαC MRP4-his_6_ was linearized using PmeI and transformed into *P. pastoris* ×33 using electroporation. Colonies containing integrated MRP4 were grown essentially as described previously for *Pichia* expression of a membrane protein.^22^ Briefly, colonies were grown in 25 mL of BMGY in sterile 250 mL flasks at 30 °C in a shaking incubator (250–300 rpm) until the culture reached an OD_600_ of 2–6. Cells were harvested by centrifugation at 3000*g* for 5 min, all BMGY was removed, and then they were washed with BMMY and resuspended in BMMY at an OD_600_ of 1.0 before being returned to the shaking incubator at 22 or 30 °C. Sterilized pure methanol was added every 24 h to a final concentration of 0.5% (v/v) methanol. Samples were taken every 24 h over a 72 h period.

### HEK293T Expression

pcDNA3.1-MRP4-his_6_ plasmid was constructed by restriction digestion of MRP4-his_6_ out of the pFastBac plasmid and ligation into a pcDNA 3.1 Zeo + plasmid. pOPINE-MRP4–3C-flag-his_8_ was made by the Oxford Protein Production Facility (OPPF, Harwell, UK). pcDNA3.1-MRP4 without a his-tag was a kind gift from Professor Susan Cole (Queen’s University, Kingston, ON, Canada). HEK293T cells were seeded in a six-well plate with 300,000 cells/well in Dulbecco’s modified Eagle’s medium (DMEM) containing 10% fetal bovine serum (FBS) and 1% penicillin/streptomycin 24 h prior to transfection. Three hours prior to transfection, the media was replaced with low-serum DMEM containing 2.5% FBS and 1% penicillin/streptomycin. For transfection, 4 µg of plasmid DNA was combined with 18 µL of 10 mM linear polyethylenimine (PEI; Polysciences, Warrington, PA) and 100 µL of reduced serum media (OPTIMEM) and added to each well. Twenty-four hours after transfection the media was replaced with DMEM containing 10% FBS and 1% penicillin/streptomycin. Samples were taken every 24 h over a 72 h period.

### Cell Lysis and Membrane Preparation

For both *Sf*9 and HEK293T, cells were harvested by centrifugation (5000*g* for 10 min) and cell pellets were resuspended in buffer 1 (50 mM Tris-HCl, pH 7.4, 250 mM sucrose, 0.25 mM CaCl_2_) containing protease inhibitors (1.3 µM benzamidine, 1.8 µM leupeptin, 1 µM pepstatin). Cells were disrupted through nitrogen cavitation at 500 psi for 15 min at 4 °C. The cell lysate was centrifuged at 750*g* for 10 min to remove cell debris; the supernatant was then ultracentrifuged at 100,000*g* for 20 min at 4 °C. The membrane pellet was resuspended in buffer 2 (50 mM Tris-HCl, pH 7.4, 250 mM sucrose) and stored at −80 °C.

*P. pastoris* cells were pelleted via centrifugation at 2500*g* for 30 min and then resuspened in buffer 3 (5.5% [w/v] glycerol, 2 mm EDTA, 100 mM NaCl, 50 mM NaH_2_PO_4_, 50 mM Na_2_HPO_4_) containing EDTA-free protease inhibitor tablets (Roche, Welwyn Garden City, UK). Cells were resuspended at a buffer (mL)-to-cell pellet weight (g) ratio of 3:1. Resuspended cell pellets were homogenized by passing them through the Emulsi Flex C3 machine (Avestin, Ottawa, Canada) three times. The homogenized cells were centrifuged at 5000*g* for 5 min, the supernatant was then centrifuged at 13,000*g* for 15 min, and finally the supernatant was centrifuged for 1 h at 100,000g. Membrane pellets were resuspended in buffer 4 (20 mM HEPES, pH 8, 50 mM NaCl, 10 % [w/v] glycerol) containing protease inhibitor (Roche) and stored at −80 °C.

### Analysis of Expression

Expression of MRP4 was monitored by Western blot. The total protein concentration of membranes was measured using a bicinchoninic acid (BCA) assay kit (Pierce, Thermo Scientific, Waltham, MA). Specified amounts (µg) of total protein were loaded on 8% sodium dodecyl sulfate–polyacrylamide gel electrophoresis (SDS-PAGE), transferred to a polyvinylidene fluoride (PVDF) membrane, and blocked with 5% (w/v) bovine serum albumin (BSA) in TBS-T (20 mM Tris, pH 7.5, 150 mM NaCl, 0.01% [v/v] Tween-20). Blots were probed with either a mouse anti-his antibody (R&D Systems, Abingdon, UK) at a dilution of 1:500 or a rat anti-MRP4 antibody (M4I-10, Enzo, Exeter, UK) at 1:100, followed by anti-mouse HRP (Cell Signaling, London, UK, 1:3000) or anti-rat HRP (Sigma, Gillingham, UK, 1:3000). All were visualized using chemiluminescence (Pierce) and a C-Digit Western blot scanner (Licor, Cambridge, UK).

### Vesicular Transport Assays

VTAs were based on the study by Reichel et al.^23^ and performed using the *Sf*9 control and *Sf*9 MRP4-expressing cell membrane vesicles from the optimized expression conditions (1 × 10^6^ cells/mL, MOI of 2, 48 h incubation). Total protein membrane protein (10–100 μg) was incubated with 10 mM ATP (plus an ATP regenerating system: 100 μg/mL creatine kinase and 10 mM creatine phosphate) or AMP and 10 mM MgCl_2_ and 1–100 μM 8-(2-[fluoresceinyl]aminoethylthio)adenosine-3′,5′-cyclic monophosphate (fluo-cAMP) (Biolog, Bremen, Germany). VTAs were conducted in buffer 2 in a 50 μL volume and incubated at room temperature for 10 min. This time period was chosen since previous kinetic studies showed it to be within the linear range.^23^ For vanadate inhibition, 500 μM sodium orthovanadate was added along with ATP. AMP-PNP inhibition was conducted by replacing the ATP with 10 mM AMP-PNP. MK571 (0.01–10 μM) was added along with ATP to measure MK571 inhibition.

After incubation, transport was stopped by the addition of 950 μL of ice-cold buffer 2. Samples were either filtered using a PVDF filter (Millipore 0.45 μM) or centrifuged at 14,000*g* for 5 min. The filter was washed with 5 mL of ice-cold buffer 2 or the pelleted vesicles washed with 1 mL of ice-cold buffer 2. The filter or pellet was solubilized with 1 mL of SDS/HEPES buffer (1% [w/v] SDS, 7.5 mM HEPES) for 15 min. The amount of fluo-cAMP transported was measured by the fluorescence signal (RFU) of the solubilized sample measured on a PerkinElmer LS55 Fluorescence Spectrometer (excitation 480 ± 5 nm, emissions 500–600 ± 20 nm). Samples were run in triplicate and an average of five scans was taken for each sample.

Data fitting for concentration curves in the VTA was performed by fitting a Michaelis–Menten, and for MK571 inhibition used a dose–response curve. Statistical analysis was performed using an unpaired two-tailed *t* test or one-way analysis of variance (ANOVA). Data fitting and statistical analysis were carried out using GraphPad Prism.

## Results

### MRP4 Expression

**Figure 1.**
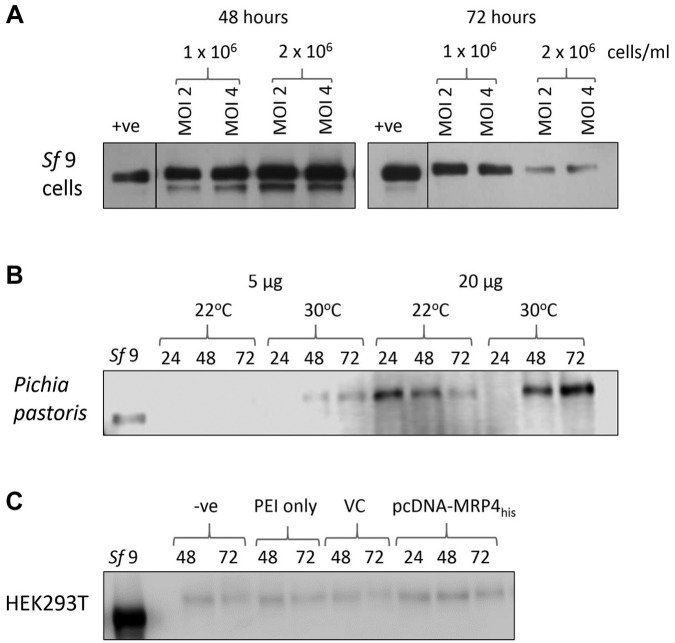
Overexpression trials for MRP4 in *Sf*9 insect cells, *P. pastoris* yeast cells, and HEK293T mammalian cells. (**A**) Western blot of MRP4 in *Sf*9 insect cell membranes after 48 and 72 h using an MOI of 2 or 4 with 1 or 2 × 10^6^ *Sf*9 cells/mL. Total protein (5 μg) was loaded for each condition. +ve represents a control sample of MRP4 expressed in *Sf*9 cells that was quantified, aliquoted, and frozen to be used as a control/standard on all Western blots to allow reliable comparison across different experiments. (**B**) Membrane expression levels in *P. pastoris* yeast cells after 24, 48, and 72 h at 22 and 30 °C. Total protein (5 or 20 μg) was loaded and compared with the *Sf*9 control expression levels (*Sf*9, 5 µg total protein). (**C**) Expression of MRP4 in HEK293T cells after 24, 48, and 72 h. Controls include untreated HEK293T cells (–ve), treatment with PEI only or with an empty pcDNA3.1 vector (VC), and the *Sf*9 control expression sample (*Sf*9). Each HEK sample contained 20 µg of total protein, whereas the *Sf*9 control contained 10 µg. Panels **A** and **B** were probed with an anti-his primary antibody and an anti-mouse HRP secondary antibody. Panel **C** was probed with an anti-MRP4 primary antibody and an anti-rat HRP secondary antibody.

The first step of the investigation was to determine the optimal conditions for MRP4 expression in each of the three expression systems, *Sf*9 insect cells, *P. Pastoris* yeast cells, and HEK293T mammalian cells. For *Sf*9 insect cell expression, the cell density, MOI, and infection period were altered. Western blots in **Figure 1A** show that the expression of MRP4 within *Sf*9 cells was successful. As reported previously, MRP4 expressed in *Sf*9 cells migrated at approximately 150 kDa.^24^ After 48 h, an increased expression was seen compared with that at 24 h (**Suppl. Fig. S1A**); however, after 72 h the expression level decreased again, possibly due to viral lysis of the cells. Increasing the cell density from 1 × 10^6^/mL to 2 × 10^6^/mL did not significantly improve the expression yield, and changing the MOI had little effect. It should be noted that a lower-molecular-weight band is also visible in several lanes, particularly those showing higher levels of expression; however, this band is not specific to these highly expressing conditions and is visible in all samples if the exposure time is increased, and has been observed previously when MRP4 was expressed in *Sf*9 cells.^24^ Therefore, the optimal expression conditions in *Sf*9 cells were 48 h with 1–2 × 10^6^ insect cells/mL at an MOI of either 2 or 4.

After successful integration of MRP4 into *P. pastoris*, the temperature and time were altered to gain the highest yield possible in shaker flasks. **Figure 1B** shows the expression of MRP4 within *P. pastoris.* Notably, the MRP4 from *P. pastoris* runs at a higher molecular weight than the *Sf*9-expressed MRP4. At a lower temperature (22 °C), the highest expression level was achieved after 24 h and then decreased over the 72 h period. At higher temperature (30 °C), the expression level increased over time, reaching the highest expression level after 72 h. The use of a 2 L bioreactor for *P. pastoris* expression was also investigated (**Suppl. Fig. S1B**); however, this gave a lower yield of MRP4 expression than the shaker flasks. The optimal conditions for *P. pastoris* expression were therefore obtained using shaker flasks at 30 °C for 24 h. However, it should be noted that this still gave a lower level of expression than the *Sf*9 cells.

Transient transfections were performed in HEK293T cells using PEI as a transfection reagent. As shown in **Figure 1C**, in contrast to *Sf*9 and *P. pastoris*, HEK293T cells express MRP4 endogenously. Transfection of the HEK293T cells with pcDNA3.1-MRP4_his_ gave only marginally increased levels of MRP4 expression. Similarly, transfection with pOPINE-MRP4-3C-flag-his_8_ led to very little overexpression of MRP4 (**Suppl. Fig. S1C**). In contrast, transfection with pcDNA-MRP4 without a his-tag gave a substantial time-dependent overexpression of MRP4 (**Suppl. Fig. S1C**).

MRP4 was successfully overexpressed in all three expression systems. However, in HEK293T cells it was only achieved in the absence of a his-tag, which would make downstream purification challenging. The yield obtained with *Sf*9 cells was higher than that achieved with *P. pastoris*. In addition, the MRP4 from *Sf*9 cells migrated at a lower molecular weight than in the other two expression systems, possibly related to the degree of glycosylation. Extensive glycosylation can be problematic for downstream structural biology; thus, this was perceived as another benefit of the *Sf*9 cell system. Therefore, the *Sf*9 expression system was taken forward to assess if the MRP4 expressed was functional.

### Vesicular Transport

**Figure 2.**
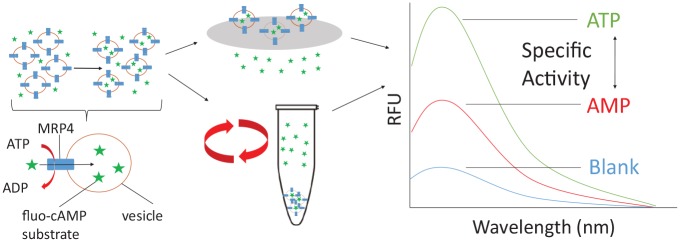
Schematic of the steps in the VTA. The first step is the incubation of the fluorescent cAMP substrate (green stars) with the membrane vesicles (orange circles) in the presence of AMP or ATP (along with an ATP regenerating system). The fluorescent cAMP is transported into the membrane vesicles via ATP hydrolysis. The vesicles are then either filtered or centrifuged to remove all excess fluo-cAMP. The vesicles are then solubilized, and the amount of fluorescent cAMP transported into the vesicles is measured on a fluorescent spectrometer. The difference between ATP and AMP is calculated giving the specific transport activity.

Finding the balance between overexpression and quality needs to be obtained. Therefore, it is vital to ascertain that the protein is functional following overexpression. To facilitate this, a fluorescent VTA was used. cAMP is a known substrate for MRP4,^25^ and this assay utilizes a fluorescent analogue of cAMP: fluo-cAMP. This substrate had previously been reported to be transported by MRP4 within renal proximal tubules and by MRP4 overexpressed in *Sf*9 membrane vesicles.^23^ By measuring the amount of substrate transported into the vesicle when ATP is present compared with AMP, the specific transport activity can be determined (**Fig. 2**).

**Figure 3.**
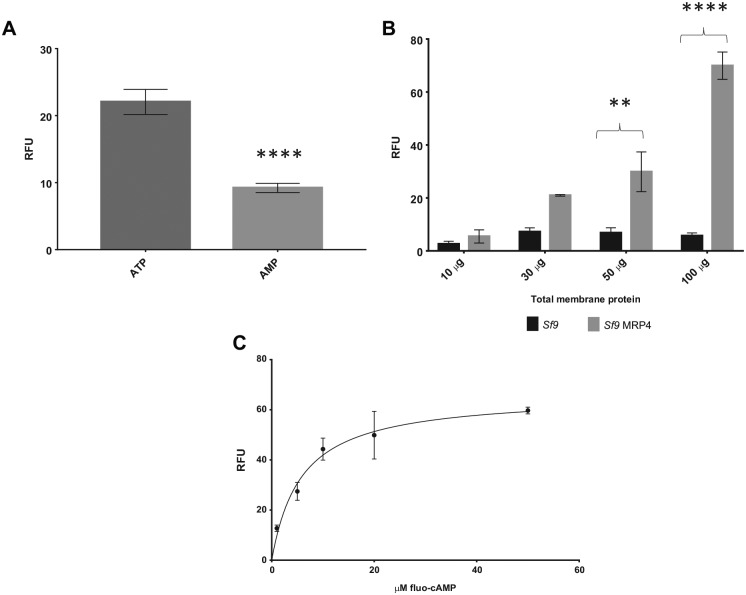
Vesicular uptake of fluo-cAMP is both ATP and MRP4 dependent. (**A**) Relative fluorescence of membrane vesicles containing MRP4 when incubated with fluo-cAMP in the presence of ATP or AMP. Total membrane protein (20 μg), with a 10 min incubation period, and 10 μM fluo-cAMP. Data are mean ± SEM, *n* = 3. Unpaired two-tailed *t* test, ****p < 0.001. (**B**) Specific transport activity of *Sf*9 control vesicles (*Sf*9) and *Sf*9 vesicles overexpressing MRP4 (*Sf*9 MRP4) using 10–100 μg of total membrane proteins and 10 μM fluo-cAMP, with a 10 min incubation time. Data are mean ± SEM, *n* = 3, two-way ANOVA, **p = 0.01, ****p < 0.001. (**C**) Specific transport activity of *Sf*9 MRP4 membrane vesicles (50 µg of protein) using 0–50 μM fluo-cAMP showing a concentration-dependent increase. Data are mean ± SEM, *n* ≥ 2, V_max_ = 64 RFU, K_m_ = 5.8 μM, Michaelis–Menten curve fitted.

**Figure 3A** shows a significant increase in the transport of fluo-cAMP in the presence of ATP compared with AMP in *Sf*9 MRP4 vesicles, showing ATP-dependent transport of fluo-cAMP. MRP4 was shown to be responsible for the transport of fluo-cAMP, as there is an increase in ATP-dependent specific activity of *Sf*9 MRP4 vesicles compared with *Sf*9 control vesicles (**Fig. 3B**). There was a positive correlation of ATP-dependent specific activity in *Sf*9 MRP4 vesicles with increased total membrane protein content, again indicating that MRP4 was responsible for the transport of fluo-cAMP, whereas the *Sf*9 control vesicles had a steady background fluorescence with increasing total membrane protein content. **Figure 3C** demonstrates a concentration-dependent transport of fluo-cAMP with a K_m_ of 5.8 μM, which is comparable to previously reported values.^23^

**Figure 4.**
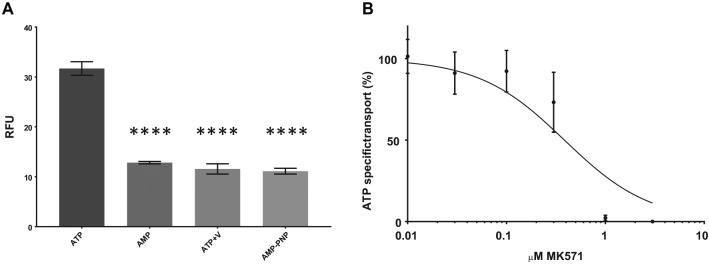
Vesicular uptake of fluo-cAMP is inhibited by nonhydrolyzable ATP analogues and MK571. (**A**) Fluo-cAMP transported (RFU) in the presence of ATP (10 mM), AMP (10 mM), ATP (10 mM) + vanadate (500 µM) (ATP + V), or the nonhydrolyzable ATP analogue AMP-PNP (10 mM). Total membrane protein (*Sf*9 MRP4, 30 μg) with an incubation period of 10 min and 10 μM fluo-cAMP. Data are mean ± SEM, *n* = 3, one-way ANOVA, multiple comparisons, ****p < 0.001. (**B**) Dose-dependent inhibition of fluo-cAMP (10 µM) transport by MRP4 *Sf*9 membrane vesicles (50 µg protein) in the presence of 0.01–5 μM MK571, with a 10 min incubation time. The percent of ATP-specific transport was measured using ATP as 100%. Data are mean ± SEM, *n* = 3, IC_50_ = 0.39 μM MK571 [inhibitor] versus normalized response curve fitted.

ATP hydrolysis is needed for the transport of substrates by MRP4, and inhibiting ATP hydrolysis should therefore inhibit transport. As shown in **Figure 4A**, in the presence of vanadate or the nonhydrolyzable ATP analogue AMP-PNP, the uptake is reduced to the same level as with AMP, indicating that ATP is the driving force behind the transport of fluo-cAMP. MK571, a known inhibitor of MRP4, was also used to demonstrate the functionality of MRP4. MK571 inhibits the transport of substrates by binding within the TMDs rather than the nucleotide binding sites like vanadate and AMP-PNP.^26^ MK571 inhibited the transport of fluo-cAMP in a concentration-dependent manner with an IC_50_ of 0.39 μM.

These results verify that MRP4 expressed in *Sf*9 cells is functional as it is responsible for the transport of fluo-cAMP in a concentration- and ATP-dependent manner and transport was prevented by inhibiting either ATP hydrolysis or substrate binding.

## Discussion

The need for good starting material is paramount in elucidating the function and structure of membrane proteins. To address this, we investigated MRP4 expression in three different systems, *Sf*9 insect cells, *P. pastoris* yeast, and HEK293T mammalian cells. All three of these systems have been successfully utilized in the past for overexpression of mammalian ABC transporters for functional and structural studies.

*P. pastoris* has been successfully used for the overexpression of mouse MRP1/ABCC1,^27,28^ mouse P-glycoprotein/ABCB1,^29^ and human TAP1/2.^30^ In this study, we found that human MRP4 could also be successfully overexpressed using *P. pastoris*. Surprisingly, the level of expression achieved was lower when using a bioreactor rather than shaker flask cultures (**Suppl. Fig. S1B**). With a bioreactor, it is possible to continuously monitor and respond to the conditions within the culture, such as oxygenation and pH; thus, it might be considered to be more optimal for cell growth. Although we were able to grow the yeast to very high cell densities within the bioreactor, this did not translate into high expression levels of MRP4. Following optimization of the shaker flask conditions, the level of MRP4 expression achieved was still lower than that obtained when using *Sf*9 insect cells (**Fig. 1B**). It might be that codon optimization of the construct could help improve this further in the future.^31^

The expression of MRP4 within *Sf*9 cells has been reported previously;^21,23,24,32,33^ however, this has predominantly been utilized for functional assays to date, rather than with the aim to develop an expression system for future purification. Here we showed that MRP4 with a his-affinity tag could be successfully overexpressed in *Sf*9 cells, and the expression level could be optimized by changing the time of infection (**Fig. 1A**). Insect cells have previously been utilized for the expression, purification, and structural study of human P-glycoprotein/ABCB1,^34^ although this used High Five (*Trichoplusia ni*) cells rather than *Sf*9 cells. Insect cells have also been proven to be especially useful for structural studies on G-protein-coupled receptors (GPCRs), which have shown a preference for S*f*9 cells.^35^

The overexpression of MRP4 in HEK cells has also been reported many times previously,^32,36–38^ but again, to date this has mainly been for the purposes of functional studies. Transient transfection of HEK cells has been carried out using the transfection reagent Lipofectamine.^36^ In this study, we have successfully shown overexpression of MRP4 in HEK cells using the much cheaper reagent PEI (**Suppl. Fig. S1C**). PEI has also been successfully utilized for the transfection of HEK cells with the related protein ABCG2.^39^ However, interestingly this only worked successfully for the untagged MRP4 construct (**Suppl. Fig. S1C**). For two different constructs containing MRP4 with a C-terminal his-tag, only minor, if any, overexpression was achieved **(Fig. 1C and Suppl. Fig. S1C**). It is unclear at this point if this could be improved with the use of an alternative transfection reagent. It is known that MRP4 contains a PDZ motif at its C-terminal, which is important for interaction with other proteins and localization within mammalian cells,^40^ and perhaps the his-tag interferes in some way. An alternative approach to transfection that has been successfully utilized for the HEK expression of other ABC transporters for structural studies is the transduction of HEK cells with a recombinant baculovirus containing a mammalian promoter.^41,42^

Notably, both the *P. pastoris* and HEK-expressed MRP4 migrated at higher molecular weights than the *Sf*9 MRP4 (**Fig. 1B,C**). It is known that MRP4 is glycosylated^36^ and *Sf*9 cells are only able to carry out simple mannose glycosylation,^6^ so this difference is likely due to differential glycosylation in the three systems. Glycosylation can be problematic for structural studies since it adds heterogeneity.

Taken together, the higher yield of MRP4, the potential lower levels of glycosylation, and the ease of scale-up led us to choose *Sf*9 cell expression to proceed with.

The next step was to check that the *Sf*9 overexpressed MRP4 was functional. Typically, function is assessed by VTAs using radiolabeled substrates; however, a fluorescent-based assay can be both cheaper and easier. It was previously shown that MRP4 can transport the fluorescent analogue of cAMP, fluo-cAMP.^23^ We found that crude membranes of *Sf*9 cells expressing MRP4 were able to transport fluorescent cAMP in an ATP-dependent manner (**Fig. 3**). The K_m_ of fluo-cAMP was found to be very similar to that found in the previous study,^23^ showing that this method is a robust way of determining the functionality of MRP4 using fluorescent analogues. Transport was also inhibited by MK571 and ATP analogues, confirming its functionality (**Fig. 4**).

During this study, both rapid filtration and a centrifugation technique were tested for separating free fluo-cAMP from the vesicles (**Fig. 2**). Rapid filtration is typically used with radiolabeled substrates; however, with the fluorescent assay particles from the filters caused an increase in light scattering, which decreased the signal-to-noise ratio. PVDF filters were better than glass fiber filters; however, the centrifugation method improved this even further, as well as increasing the efficiency of the transport assay.

In conclusion, we have successfully demonstrated functional overexpression of MRP4 in *Sf*9 cells that can now be taken forward for solubilization and purification to enable mechanistic and structural studies.

## Supplemental Material

DS_DISC867070 – Supplemental material for Functional Expression of Multidrug Resistance Protein 4 MRP4/ABCC4 Click here for additional data file. Supplemental material, DS_DISC867070 for Functional Expression of Multidrug Resistance Protein 4 MRP4/ABCC4 by David Hardy, Roslyn Bill, Anass Jawhari and Alice Rothnie in SLAS Discovery
