# Supplementary material for: Functional Expression of Multidrug Resistance Protein 4 MRP4/ABCC4
Source: SLAS Discov. 2019 Aug 5;24(10):1000–8. doi: 10.1177/2472555219867070 (PMC6873218; doi:10.1177/2472555219867070)
Supplement: DS_DISC867070 – Supplemental material for Functional Expression of Multidrug Resistance Protein 4 MRP4/ABCC4 [file DS_DISC867070.pdf]

Functional expression of MRP4/ABCC4

David Hardy[1,2], Roslyn Bill[1], Anass Jawhari[2] & Alice Rothnie[1*]*

Supplementary Information

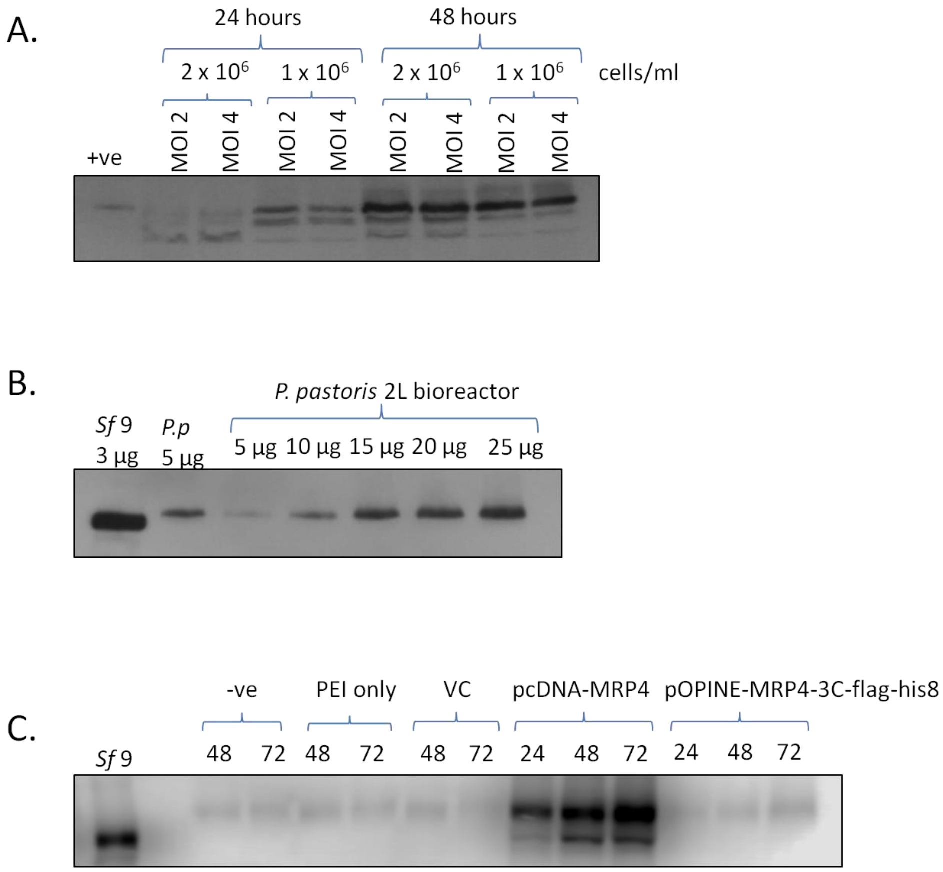

Supplementary Figure 1: Overexpression trials for MRP4 in *Sf*9 insect cells, *P. pastoris* yeast cells and HEK293T mammalian cells. A; Western blot of MRP4 in *Sf*9 insect cells (whole cell lysates) after 24 and 48 hours using an MOI of 2 or 4 with 1 or 2 x 10^6 Sf 9 cells/mL. +ve represents a control/standard sample of MRP4. B; membrane expression levels in *P. pastoris* yeast cells grown in a 2L bioreactor in comparison to *P. Pastoris* grown in shaker flasks. Specified amounts (µg) of total protein were loaded, and compared to control *Sf*9 expression levels (*Sf*9, 3µg total protein). C; Expression of MRP4 in HEK293T cells after 24, 48 and 72 hours using the pcDNA3.1-MRP4 un-tagged construct or the pOPINE-MRP4-3C-flag-his_8 construct. Controls include untreated HEK293T cells (-ve), treatment with PEI only or with an empty pcDNA3.1 vector (VC) and an *Sf*9 expressed MRP4 positive control (10µg). All HEK samples contain 20µg total protein. A & B were probed with an anti-his primary antibody and an anti-mouse HRP secondary antibody. C was probed with an anti-MRP4 primary antibody and an anti-rat HRP secondary antibody.
